# Supplementary material for: Characterization of the Bacterial Communities Inhabiting Tropical Propolis of Puerto Rico
Source: Microorganisms. 2023 Apr 26;11(5):1130. doi: 10.3390/microorganisms11051130 (PMC10221019; doi:10.3390/microorganisms11051130)
Supplement: Supplementary file 1 [file microorganisms-11-01130-s001.zip › microorganisms-2335020-supplementary.pdf]

Table S1. Antimicrobial activity of alcoholic propolis extracts against tester bacterial strains. AMP= Ampicillin; TET = Tetracycline; #4dH2O = #4 aqueous extract; EtOH = 70% ethanol; dH_2O = distilled water; - = no inhibition (growth); -/+= partial inhibition; += complete inhibition (no growth); nm = not made.

**Tested solutions**		**Tester strains**				
		E. coli	***A. baumannii***	***B. subtilis***	***E. faecalis***	***S. aureus***
AMP		+/-	+	++	+	++
TET		+	+/-	++	+/-	++
LUCIA	Tq	+/-	+/-	+	+/-	+
	1:2	nm	nm	+	-	-
	1:4	nm	nm	+/-	-	-
	1:8	nm	nm	+/-	-	-
AKIRA		+	+/-	+	+	+/-
CUQUITA		+	+	+	+	+
DIABLA		+	+	+	+	+
DUKE		+/-	+	+	+	+
FLORENCIA		+/-	++	+	+	+
#1		+/-	++	++	+	++
#2		+/-	++	+	+	++
#4	Tq	+/-	+/-	++	++	++
	1:2	nm	nm	+	+/-	+/-
	1:4	nm	nm	+	+/-	+/-
	1:8	nm	nm	+	+/-	+/-
#4 H_2O		nm	-	-	-	-
#8		+/-	++	++	+	++
#11		+/-	++	++	+	++
#13		+	+	+	+	+
EtOH	Tq	+/-	+/-	-	+/-	+/-
	1:2	nm	nm	-	-	-
	1:4	nm	nm	-	-	-
	1:8	nm	nm	-	-	-
dH_2O		-	-	-	-	-

Table S2. Linear discriminant analysis (LDA) effect size (LEfSe) on bacterial communities inhabiting propolis samples from Yauco and Arroyo areas, with different dominant vegetation. NA = Not Available.

**Phylum**	**Family**	**Genus**	**Dominant Vegetation**	**Area**	**LDA**	**p-value**
Proteobacteria	Rhodocyclaceae	Dechloromonas	Dry Forest	Yauco	2,077	0,019
Proteobacteria	Enterobacteriaceae	NA	Dry Forest	Yauco	2,053	0,019
Proteobacteria	Sphingomonadaceae	NA	Dry Forest	Yauco	2,225	0,019
Actinobacteriota	Pseudonocardiaceae	Pseudonocardia	Dry Forest	Yauco	2,057	0,019
Proteobacteria	Rhodocyclaceae	Dechloromonas	Dry Forest	Yauco	2,246	0,019
Proteobacteria	Rhodocyclaceae	NA	Dry Forest	Yauco	2,056	0,034
Proteobacteria	Burkholderiaceae	Burkholderia-Cab. Paraburkholderia	Dry Forest	Yauco	2,071	0,045
Actinobacteriota	Pseudonocardiaceae	Pseudonocardia	Dry Forest	Yauco	2,881	0,045
Proteobacteria	Enterobacteriacea	NA	Dry Forest	Yauco	2,686	0,047
Proteobacteria	Burkholderiaceae	Burkholderia-Cab. Paraburkholderia	Dry Forest	Yauco	3,591	0,047
Proteobacteria	Rhodocyclaceae	Dechloromonas	Dry Forest	Yauco	2,077	0,019
Proteobacteria	Rhizobiaceae	NA	Moist Forest	Arroyo	2,195	0,005
Proteobacteria	Xanthomonadaceae	Pseudoxanthomonas	Moist Forest	Arroyo	2,814	0,007
Actinobacteriota	Brevibacteriaceae	NA	Moist Forest	Arroyo	2,809	0,007
Bacteroidota	Sphingobacteriaceae	Sphingobacterium	Moist Forest	Arroyo	3,036	0,007
Actinobacteriota	Brevibacteriaceae	Brevibacterium	Moist Forest	Arroyo	2,809	0,007
Proteobacteria	NA	NA	Moist Forest	Arroyo	3,294	0,009
Bacteroidota	Sphingobacteriaceae	NA	Moist Forest	Arroyo	3,220	0,009
Proteobacteria	Enterobacteriaceae	NA	Moist Forest	Arroyo	3,199	0,009
Proteobacteria	Xanthomonadaceae	NA	Moist Forest	Arroyo	3,225	0,009
Proteobacteria	Sphingomonadaceae	Sphingomonas	Moist Forest	Arroyo	2,427	0,019
Proteobacteria	Rickettsiaceae	Ac37b	Moist Forest	Arroyo	2,336	0,019
Proteobacteria	Rickettsiaceae	Ac37b	Moist Forest	Arroyo	2,336	0,019
Proteobacteria	Rhizobiaceae	Allorhizobium-Neorhizobium-Pararhizobium	Moist Forest	Arroyo	2,516	0,026
Proteobacteria	Sphingomonadaceae	Sphingobium	Moist Forest	Arroyo	2,108	0,026
Proteobacteria	Erwiniaceae	NA	Moist Forest	Arroyo	3,321	0,028
Proteobacteria	Xanthomonadaceae.	Stenotrophomonas	Moist Forest	Arroyo	2,956	0,028
Proteobacteria	Beijerinckiaceae	1174_901_12	Moist Forest	Arroyo	2,157	0,034
Bacteroidota	Weeksellaceae	NA	Moist Forest	Arroyo	2,662	0,047
Bacteroidota	Weeksellaceae	Chryseobacterium	Moist Forest	Arroyo	2,651	0,047
Proteobacteria	Rhizobiaceae	Allorhizobium-Neorhizobium-Pararhizobium	Moist Forest	Arroyo	2,695	0,047
Bacteroidota	Flavobacteriacea	NA	Moist Forest	Arroyo	2,185	0,047
Proteobacteria	Pseudomonadaceae	NA	Moist Forest	Arroyo	3,074	0,047
